# ﻿Workflow designs can facilitate sample management for student-research teams doing taxonomic resource development with Trichoptera

**DOI:** 10.3897/zookeys.1263.150420

**Published:** 2025-12-10

**Authors:** Patina K. Mendez

**Affiliations:** 1 Department of Environmental Science, Policy and Management, University of California, Berkeley, 130 Mulford Hall #3114, Berkeley, C.A. 94720, USA University of California Berkeley United States of America

**Keywords:** Benthic macroinvertebrates, caddisfly, sample processing, species pages, student research, taxonomy

## Abstract

Undergraduate research groups can face challenges in their involvement in multi-year or long-term research projects in maintaining momentum for projects, keeping consistency among student researchers, minimizing errors, and retaining institutional knowledge. Management of a large student group with a wide range of projects requires workflows that streamline sample processing for both multi-year projects and developing localized Trichoptera taxonomic identification resources for a specific study region. This paper presents workflows for undergraduate researchers to facilitate (1) sample sorting and identification of adult caddisflies from 4 years of pan trap samples, and (2) developing taxonomic “species pages” that include species description and illustrations for 300+ species of California caddisflies. For the past ten years, almost 80 undergraduate members of the research team have contributed to museum and ecological studies to gain skills recognizing adult Trichoptera, using keys, and gaining familiarity with taxonomic resources such as species descriptions. Undergraduate researchers participate for 2–3 semesters, learn to collaborate, and develop research skills to identify samples. Research team activities have contributed to a 4-year dataset of monthly adult activity in an intermittent stream that will be used for continuing research questions. Moreover, they have compiled a resource for species-level identifications for California caddisflies.

## ﻿Introduction

Undergraduate research opportunities are often the first experiences for students to actually “do science” outside of their coursework. However, learning to be a scientist takes time, and requires access to research experiences and opportunities to follow interests. This “scientific-identity building process” occurs after multiple or long-term research opportunities (e.g., 3 or more semesters, Thiry and Laursen 2011; Linn et al. 2015). For entomologists, these research apprenticeships are standard practice to facilitate the transfer of knowledge and techniques used by researchers and practitioners to students in respect to field collection, research approaches, specimen curation, and laboratory practices. These learning experiences and relationships are critical to fostering student interests and skills and for training future entomological workers.

Research laboratories that primarily host undergraduate students in learning experiences can face challenges in undertaking multi-year or long-term research projects. At smaller institutions, constraints on faculty time to serve as student mentors limits research opportunities for undergraduates. In contrast, at large institutions, even with graduate students available as mentors, demand for research experiences for students far exceeds available positions. Although a rewarding experience for mentors, time constraints and turnover of undergraduate researchers may reduce project momentum, requiring workflows and documentation to keep project consistency, reduce student training demands, minimize errors, and retain institutional knowledge. Fortunately, research opportunities can be scaled by supplementing faculty-based mentoring with near-peer mentoring in a collaborative research environment that benefits novice and experienced students (Tenenbaum et al. 2014). Furthermore, framing the research environment as collaborative can provide “kindness cues” that signal both inclusion and belonging for the students; these cues align with values of students from backgrounds historically underrepresented in STEM toward increasing student persistence (Estrada et al. 2018).

In this paper, I present two example workflows centered on studies of caddisflies (Insecta: Trichoptera) in California to facilitate small and large team collaboration for (1) management of invertebrate samples for sorting and identification processing, and (2) creating taxonomic identification resources such as digital “species pages”. These workflows allow for synchronous, collaborative work in an undergraduate student learning environment.

## ﻿Team Caddis students and learning activities

Team Caddis is an undergraduate research group that works on small-scale, iterative, and collaborative ecological and faunistic studies at the University of California, Berkeley. Team members include novice researchers, students with intermediate levels of experience, and senior thesis students. I emphasize a collaborative research philosophy that promotes team-based research projects, inclusive practices, and open communication policies. To provide more research opportunities, I use a near-peer mentoring model to manage student projects and participation. Many students express that these values and practices encourage them to apply to the research group.

Students join Team Caddis through the Sponsored Projects for Undergraduate Research (SPUR) program in the Rausser College of Natural Resources, the Undergraduate Research Apprenticeship Program (URAP), or for a senior thesis. SPUR and URAP students often join the lab for their first research experience for 3 hours/week for course credit. Some students have taken General Entomology, but most lack experience in insect identification. Students often participate for 2–3 semesters, or may leave to join other labs more aligned with their career interests (e.g., wildlife, air pollution, molecular biology). In any one semester, Team Caddis has ~12 undergraduate researchers: 2–6 novice within the first year of research experience, 3–5 intermediate students who train and serve as near-peer mentors to novice students and begin more independent projects, and 3–4 senior thesis students conducting independent research. I lead all these groups. All students are invited to gain field experience through assisting in fieldwork for senior thesis projects. Over the past 10 years, almost 80 students have participated in research with Team Caddis, but because students participate for more than one semester, the total number of semester-long research experiences that have occurred is closer to 175.

As part of Team Caddis, students learn to use microscopes and Linnean taxonomy, recognize caddisflies, use taxonomic keys for family identification of adult caddisflies, and may learn to use larval keys to identify benthic macroinvertebrates. Students use Wiggins (2005) to identify adult Trichoptera to family level, Merritt et al. (2019) (including older editions) and McCafferty (1998) to identify benthic macroinvertebrates. Species-level identifications remain rare as we have only recently developed the “species pages” described in the second workflow in this paper.

Senior thesis students conduct year-long research projects focused on aquatic ecology or entomology. Most students are supported by me in developing their study designs, research skills and scientific writing through required coursework in their major. Alternatively, I may teach them these skills alongside data collection activities. Senior thesis projects include surveys of benthic macroinvertebrates of urban and recreational streams, impacts of contaminants such as mercury mining or microplastics, and/or terrestrial projects on behavior (e.g., beetle responses to artificial light at night, pollination studies). Some projects leverage or expand upon existing material including the use of wing morphometrics to make male-female species associations or toward developing identification characters.

Learning goals beyond identification include skills in fieldwork and collaboration. All students are invited to participate in fieldwork for senior thesis projects to learn how to use field equipment and to conduct protocols from the Surface Water and Aquatic Monitoring Program (SWAMP, Ode et al. 2016), which include rapid bioassessment protocols for benthic macroinvertebrates and physical habitat measurement such as pebble counts, stream flow and discharge measurements, water chemistry, canopy cover and visual habitat assessments. Senior thesis students learn project management skills and time management, develop full fieldwork plans and modify protocols, and manage fieldwork activities and sample processing. They also mentor other students assisting in processing samples for senior projects and serve as role models for more advanced research activities. I participate in mentoring the students in the lab through check-ins and through fieldwork. Most often I work directly with students in the laboratory to teach them to use the keys, explain morphology, and check identifications.

## ﻿Sample sorting and identification workflows

### ﻿Motivation for developing the sorting and identification workflows

**Figure 1.**
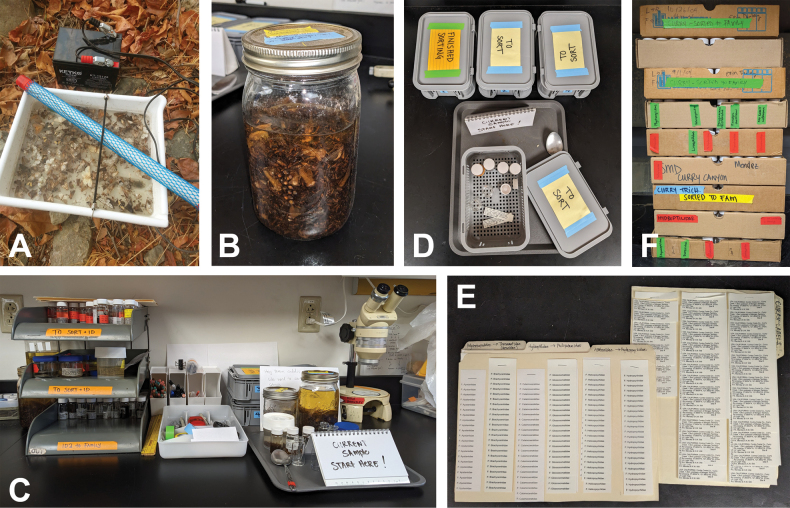
Setup for sorting and identification of adult Trichoptera. The setup for student workers includes: A. Light trap sample in the field; B. Large bulk sample from one trap; C. Lab setup for team members; D. Family-level ID sorting setup; E. Pre-printed family labels (left) and fillable locality labels (right), and F. Scintillation vial flats of caddisflies identified to family. Supplies for students (in the center of panel c) include forceps and manipulators, pens with archival pigment ink (Pigma) and pencils, strips of label paper (Resist-all), scissors, prepared (pre-printed and fillable) locality labels and family labels stapled to a file folder, and extra empty vials.

The motivation for a sample management workflow was driven by a 4-year study from 2015–2019 of a small, intermittent stream in northern California (Curry Creek, Contra Costa Co., CA U.S.A., 37°51'59.8"N, 121°52'56.3"W). Researchers at the University of California, Berkeley (Patina Mendez, Kipling Will, Casey Hubble, and various field volunteers) deployed 4 UV light pan traps each new moon along a 1-km stream segment of Curry Creek, an intermittent tributary to the more perennial Marsh Creek (Fig. 1A). Lights were set before dusk and ran overnight to collect attracted insects into a pan trap filled with ethanol and propylene glycol. Caddisflies appeared in the pan traps in most months, except for cold and rainy winter months. The pan traps generated a tremendous amount of Trichoptera and other material during the summer months when the Curry Creek was dry. These adults may originate from isolated but hydrologically connected pools in the stream channel, dry-season aestivating adults (Coleburn 1984), or caddisflies dispersing along the dry channel (Graham et al. 2016) from Marsh Creek.

### ﻿Research questions and challenges

For this study, Team Caddis asks the question, “What caddisflies are present at this site and how does the flight phenology correlate to year-to-year variability in precipitation within the California Mediterranean Climate?” Research aims from this material included developing species-level taxa list of Trichoptera from the area that includes an annual phenology of flight times by year for the species, in the style of Erman (1989). The total number of samples (~150× 1-L Nalgene bottles) necessitated that many undergraduate researchers would participate in the research to sort, identify, and count material in the samples. Progress on sorting and identification was interrupted by campus closure during the COVID-19 pandemic in 2020. Because the project was long-term, and the learning curve for picking and identifying caddisflies was high, I created a workflow for sample management leveraging near-peer mentoring among students in the lab group.

### ﻿Workflow design

The guiding principle of the workflow design is to maintain integrity of the samples (i.e., avoid mixing up samples) at all phases of the project during the: (1) picking and sorting phase from the bulk field sample, (2) family level ID phase from the Trichoptera material, and (3) species-level identifications and counting individuals. To maintain sample integrity among students within a scope session and between sessions, all students work on a common sample until it is completed. For example, during the picking and sorting phase of a bulk field sample, one of the large sample jars is designated as the current sample that will be worked on until it is complete. For family level IDs for Trichoptera, only one small sample jar is active. These activities are facilitated by designated sample areas, signage, and communication in the lab group. To communicate the workflow, I explain it to each student and then send out a short video (2 mins, recorded on my phone) that fully explains the system.

### ﻿Picking and initial sort of bulk material

Students sort the designated bulk sample (Fig. 1B) at a communal table by eye, using a table-mounted magnifying glass 5× on an arm, or with a dissecting microscope. They separate all caddisflies to a “caddisfly” scintillation vial (or larger for some samples) as a first-level sort and add a pre-printed family and collection labels to each sample. Some non-target material (e.g., mayflies, stoneflies, and other aquatic groups, beetles, and some other terrestrial taxa) is retained in separate vials and labeled. Moths are moved to their own petri dish for eventual disposal. Students check each other’s “leftovers” including the moths for caddisflies, and then I do a final check of the leftover material for commonly missed groups such as Hydroptilidae (because of their moth-like appearance). The process of multiple checks by different operators of the remaining material from bulk field samples is critical to avoid operators from missing material to exclude small or rare taxa (Haase et al. 2010).

### ﻿Family-level sorting and identifications of samples

Family-level sorting and identifications from the bulk samples may take several laboratory sessions and days to complete. I organize samples for the family-level sort on a stackable paper tray before designating them for sorting (Fig. 1C). This area includes two shelves that fit the scintillation vials labeled “To Sort and ID” and one shelf for “ID’d to Family”. “To Sort and ID” samples are moved to the student workflow baskets, and “ID’d to Family” samples are stored on the shelf until they are moved to the scintillation flats (100 vials) to group vials by family (Fig. 1F). The scintillation vial flats will be identified to species by working one family at a time after all family level IDs are complete.

To have samples ready for students to study, I created a system that designates the current active sample, the next samples to sort, and finished samples (Fig. 1D). Each sample is placed in a basket with prepared locality labels that match the sample, and a set of empty vials. Each basket has a loose lid that has “To Sort” on one side and “Finished Sorting” on the other side, so that the lid can be flipped depending on the status of the sample. One area, in this case a tray, has a sign with “Current Sample” and includes one sample basket and any notes about what needs to be done. All students work on the “Current Sample” using the adult family-level key from Wiggins (2005) and ask each other for help with difficult families. As they make identifications, they create a separate vial for each family, using the prepared locality and family labels (Fig. 1E). When the working team completes a sample, they flip the basket lid to “Finished Sorting” and move a new sample into the “Current Sample” area. Because the lids visibly reflect the status of the samples, I can quickly identify progress: for finished samples, I can check labels and ethanol levels, rotate out the finished samples, and rotate new samples into the workflow.

### ﻿Benefits of this workflow design

The workflow design allows students to work as a team toward a larger project goal, see progress, and have clear guidelines to maintain sample integrity. In past projects, students often worked on their own separate sample, increasing the bench space needed for storing active samples. Students did not always complete samples before the end of the semester, resulting in “orphaned” samples requiring completion and evaluation for accuracy. The collaborative workflow design instead allows for senior members to teach lab workflows to new members, for members with overlapping schedules to pass information about the sample, and for team members to help each other with challenging identifications in a near-peer mentoring relationship. Improvements to this workflow design would be a log sheet for students to record which sample they worked on for their work sessions. This would aid tracking of questions, provide information on who worked on a sample with identification errors and to estimate handling time required for processing samples.

Some students have developed their own identification guides for family-level identifications of Trichoptera using Google Slides by taking photos through the microscope and noting characters to help them learn identifications. Because the students sometimes get bored by making only family IDs, they express joy at being presented with a 1L jar full of, for example, the caddisfly family Sericostomatidae, other caddisflies, and bycatch for bulk sorting. They find the bycatch that includes tiny flies and small leafhoppers very interesting. Two students have used material from large samples for their senior thesis projects in which they imaged caddisfly wings and conducted morphometric analysis of the venation of fore- and hind wings. Over 40 undergraduate students have participated in sorting and identifying caddisflies for this project since 2015.

### ﻿Ongoing work and challenges

As of 2025, nearly all the material is identified to family (Fig. 1F, ~700 vials), and the team is ready to begin making identifications to the genus and species level using available keys and “species pages” which include species descriptions assembled by Team Caddis. Because these family-level identifications are made by learners I expect that some of the family-level identifications will be initially incorrect. When training the students, we talk about the learning process for identifications and how it is and iterative process: (1) the first few times through the keys they may make mistakes, (2) after viewing more material from samples, they will see other examples of characters and revise their understanding, (3) after we make the initial sort of family, we will go through each family individually again much more closely to check them, and (4) when we make genus and species determinations, we will again make a closer examination of the material. Because sample and label associations are maintained, we will be able to find and correct these errors when we work through the material for the final species-level determinations, sex determinations and counts.

There will be some challenges for students in making the species identifications because students will need to develop more technical skills in specimen preparation. Making species-level identifications for Trichoptera requires “clearing” genitalia to make external and internal cuticular structures visible to compare through keys and species descriptions. The standard practice requires separating the abdomen from the insect body and placing it in a vial with hot lactic acid or KOH and rinsing the material at the appropriate time (see Holzenthal and Andersen 2004). In addition, the most abundant material includes tens of thousands of Sericostomatids (*Gumaga*) which include potentially cryptic species recognized as adding challenges in ecological studies in California (Jackson and Resh 1998).

## ﻿Species pages workflows

### ﻿Motivations for developing a digital species pages resource for California Trichoptera

The motivation for creating species pages workflows is based on the need to identify California caddisflies from light-trap samples to species. “Species pages” are one to several page document or web page that includes illustrations, taxonomic description, and identification notes that can be useful for researchers making identifications or working on taxonomy. Several resources exist for species-level identifications for California caddisflies. Burdick (2010) assembled a digital, unpublished document with a California species list and species pages with multi-view illustrations of males of most species by Shannon Bickford and included about ~3000 records primarily from the California Academy of Sciences Trichoptera Collection. However, this work does not include any accompanying taxonomic identification or descriptive information. Although dated, Denning (1956) earlier provided keys that can be helpful for some species as a starting point.

Mendez et al. (2019) developed a preliminary species list for the caddisflies of California with a conservative estimate of 333 species, using Denning (1956), the *Distributional Checklist of Nearctic Trichoptera* (Rasmussen and Morse 2018), Burdick’s (2010) list, and records from GBIF and other natural history museums. Resh and Mendez (2022) later updated this list with additional records from California natural history museums for a potential 358 species. However, even with these resources, readily accessible descriptive information for making species-level identifications in California is still lacking for some males and largely absent for females.

Team Caddis members had opportunities to participate in research through remote work and assist with digitation efforts of taxonomic resource and museum records. For example, 11 Team Caddis members worked on digitizing records of scanned fluid-based material from the Essig Museum of Entomology at the University of California, Berkeley using Google Forms. Students entered > 400 records in ~20–30 hours (3–5 mins per entry). After those were completed, students digitized records from Burdick (2010) and eight students entered > 3000 records from Feb-Dec 2019. Both projects involved log sheets to track which student entered each record to reduce duplication of efforts and track progress.

Although already planned but not started, much of the work to develop species pages occurred during the COVID-19 pandemic when the University of California, Berkeley, campus was closed to all non-essential research activities. I aimed to keep research opportunities available for undergraduates by moving all work to digital activities in an online virtual lab space to keep connection with students. The team began working remotely on the species pages in Oct 2020 and finished in Aug 2022.

### ﻿Species pages components

Each species page is designed to be a stand-alone resource with one species per document (often longer than one printed page) with illustrations, species descriptions, and identification annotations. Construction of species pages is a common practice for making identifications, usually by copying or scanning journal articles and arranging illustrations and descriptive information on one or more pages. These pages can also include supplemental materials such as annotations, photographs, additional illustrations from other publications, and expanded descriptions by other researchers. When systematically compiled for all species in a group or region, they represent an incredible resource, especially if available online such as *Trichoptera Fennoscandinavica* (Tobias and Tobias 2014) and *Trichoptera Africana* (Tobias and Tobias 2012).

### ﻿Workflow design

The workflow design aimed to develop the pages in two phases so that all pages created would have an equivalent level of progress, beginning wide and shallow in the initial phase, and deepening the level of information in the second phase. The team worked exclusively in Google Docs and Drive while using available resources such as the *Trichoptera World Checklist* (TWC; Morse 2025), the *Trichoptera Literature Database* on Zotero (TLD; Holzenthal and Mendez 2022) and literature available through the *Biodiversity Heritage Library* (BHL) or accessed through online journals. I made detailed instructions on how to complete each page with examples and set formatting guidelines for page and file names.

#### ﻿Phase 1: Species information and reference material

**Figure 2.**
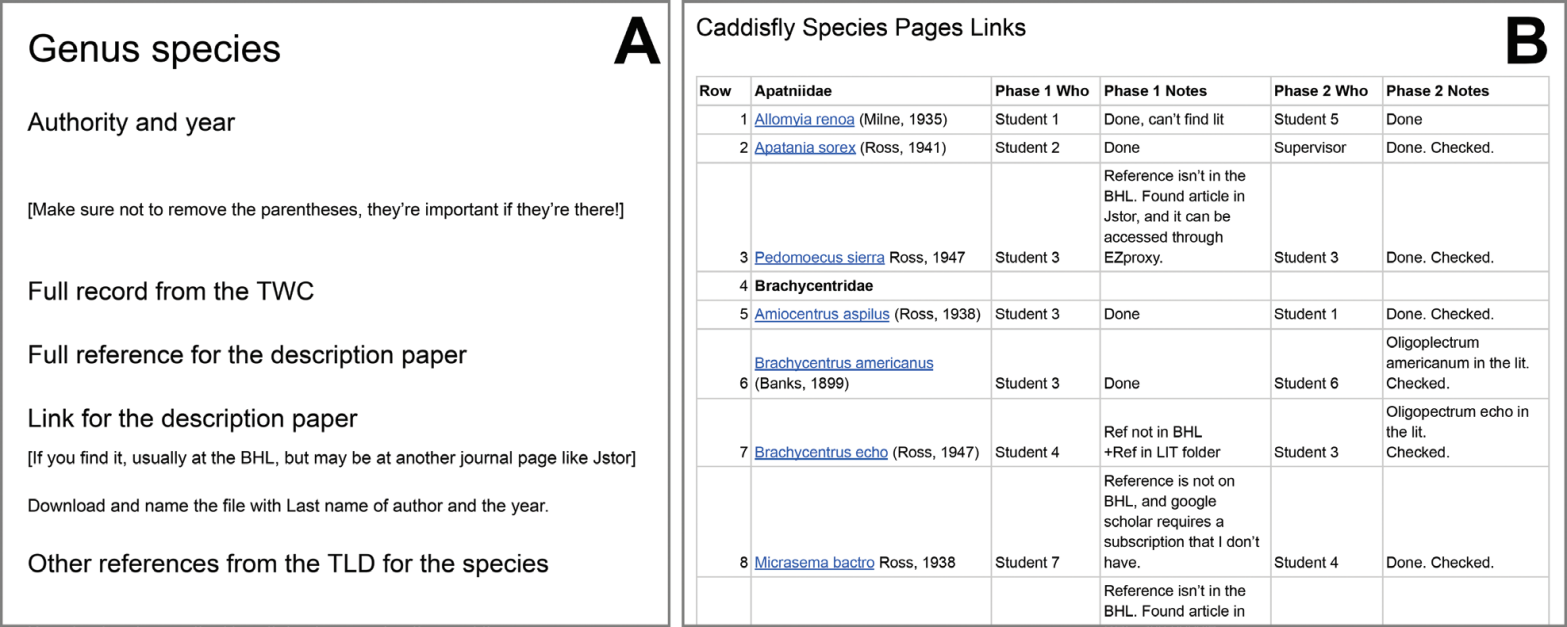
Examples of Google Docs for creating species pages. Students made a copy of the A. Species page template for each species and then B. Linked it to the appropriate species name on the tracking page. Note: Student names have been anonymized.

Phase 1 aimed to gather the basic information and reference material. I created a table in a Google Doc with species listed in alphabetical order by family (Fig. 2B) based on the species list in Mendez et al. (2019). Each row in the table included each species with the authority (incl. year) and columns for: (1) who worked on the page, (2) notes from the student, and (3) notes from me so I could respond to questions. To begin work, students would “claim” a species to work on by adding their name to the column, copy the species page template, rename it with the species name and authority, and hyperlink the page to the name in the main table for easy access.

The species page template (Fig. 2A) included a title area for the genus and species and the following headings: (1) authority and year, (2) full record from the TWC, (3) full reference to the description paper, (4) link for the description paper, and (5) other references from the TLD for the species. Once the page was created and linked, the students populated the page by looking up the species name in the TWC, checked to see if the name was valid, and copied the record into the species page. Next, students retrieved the full bibliographic information for the species from the TLD using the author name and year and added it to the document. Then they searched and downloaded a PDF of the literature that was primarily available from the BHL and renamed the file. If they had challenges, the students left notes on the worksheet to which I could respond. Nine students worked on Phase 1. We completed Phase 1 as a team before moving on to Phase 2.

#### ﻿Phase 2: Capture of species descriptions

Phase 2 aimed to capture the species description and associated illustrations, and to transcribe the species description. I copied the table from the Phase 1 Google Doc and added two columns for the name of who retrieved the description and any notes (Fig. 2B). Students again “claimed” species to work on and completed the new tasks by revising the existing species page file (Fig. 2A). For each species, students captured two screenshots and pasted them into the Google Doc under the link for the description paper: (1) the species description and (2) all illustrations. I required students to capture the original description as an image so that I could review it for transcription errors and preserve special characters. They also assembled a separate composited image file that included both the description and the illustration, saved it as a .png for reference and recorded the filename on the species page. Finally, students transcribed the species description and figure legends into text from the image snapshot. Eight students worked on Phase 2.

### ﻿Benefits of this workflow design

Team Caddis completed the 330+ species pages during a period of approximately a year and a half with each team member working 3 h per week to build the pages. This workflow design allowed each person working on the project to be able to work separately on files without duplicating effort because the file was “checked out” by noting it on the main list. The main list was visible to all team members at the same time rather than allocating lists of species to each team member. Each team member could see the overall progress on the project in real-time, and no pages were left incomplete. For this project, we all worked together in a virtual online space where students could ask questions in real time. Although we were in an online environment, students were still pretty quiet and the space was not as social as the lab. Students did say that even if it was quiet, they still experienced community and support during the pandemic through the online research space. Across the two phases, nine students participated in developing species pages.

Students enjoyed reading species descriptions and looking at the illustrations! Many gained an appreciation for taxonomic work, the illustrations, descriptive details and were able to begin to see the differences between species. One student used the species descriptions and illustrations as the basis for her senior thesis project where she catalogued and evaluated the body parts described for males and females and the quality of illustrations.

### ﻿Ongoing work and challenges

Work on this project will continue, ideally by adding images from Burdick (2010) to complement and enhance visual resources for identifications and to fill gaps where original species descriptions did not include images. To make descriptions more comparable and augment them with more descriptive information for identification, the descriptions need to be further extracted into standard tables by body part, and I will need to develop a system for distinguishing original description information from annotation. Using tables for grouping and comparing information may allow for better internal organization.

Challenges for internal use result from the high number of individual documents that make it difficult to flip between species pages. However, new features in Google Docs include using a “tab” interface (like the worksheet tabs in Google Sheets) that can allow a user to create a tab for each species. These tabs could be ordered such that each species is grouped by genus within a larger “family” Google Doc. The tab organization would reduce the number of files to 20 for California but may require downloading each tab separately for backing up. An important caution when using Google Drive for collaborative files: each file is “owned” by the creator of the file, which means if a student creates a file, you may lose access to it when they graduate, if the student deletes it, or the institution may delete orphaned files. To prevent this issue, students must “transfer ownership” using the Google share tools to the permanent maintainer of the lab resource. A best practice may be for you to create the initial files or make a copy of the final student-created file so that you are the owner. The resource should be backed up regularly as a .zip file for archiving or by using Google Takeout to download large numbers of files.

## ﻿Other considerations for undergraduate research spaces

As with any research space, time and space are often limiting resources requiring intentional strategies to maximize learning and research opportunities for students. Most students are scheduled into the laboratory for one 3-hour work session per week. This schedule is optimized to include overlaps between students to build a community with ~3 students in the lab at any one time (limited by the number of scopes). Senior thesis students have much more flexible schedules and are scheduled in for more lab time to work through their samples. All dissecting scopes, lab-provided sorting tools, taxonomic keys and books are designated as community resources. Scope stations are to be kept clean and counters clutter-free, which is a noble goal that we continually work towards by creating designated areas for work and sample storage.

Given the number of samples that each project can generate (and the space they take), each senior thesis student is assigned a 5-gallon bucket with a lid labeled with their name to keep samples and other supplies specific to their project. The benefits of buckets are that they can be kept under tables or can be stacked in the corner of the lab and are easy to move to make space. These buckets may contain zip-locks or mason jars with bulk field samples or supplies for water-testing. After sorting, students store scintillation vials in the bucket or in a flat. At the end of projects, buckets and vials can be cleaned out and used for the next student.

To reduce stress and mistakes in the field, Team Caddis has developed collections of methods, resources (e.g., lab and field protocols), and documented data collection processes. We have created Google slide decks with embedded YouTube videos for field methods and identifications that students can watch before doing fieldwork. We have standardized datasheet formats and fields for field collections and have developed full fieldwork plans that include division of labor in the field, packing lists to prepare for fieldwork, and designated and labeled field bags for equipment storage. Students are also required to keep a project management Google Doc that outlines their research questions, data collection methods, data analysis methods, and lists resources and meeting notes. Upcoming improvements to resources will be in developing standardized identification datasheets for benchwork, sample processing logs, and data entry workflows and forms.

## ﻿Conclusions

Workflows can benefit most research projects but have high returns for undergraduate research teams. These workflows can reduce errors through clear and detailed sample management communicated across team members. Students working in teams can, over time and with clear instructions, develop extensive resources that facilitate future work in the research group. Novice researchers can have a high impact for sample processing and resource development and near-peer mentoring by intermediate and senior thesis students can teach advanced skills in field methods or identification of samples. In terms of the projects, students have helped to assemble an extensive 4-year dataset of adult activity, developed an extensive resource for species identifications, and laid the groundwork for future projects leveraging both the material and resources.

## ﻿Additional information

### Use of AI

No use of AI was reported.

### Author ORCIDs

Patina K. Mendez https://orcid.org/0000-0002-9348-2998

### Data availability

All of the data that support the findings of this study are available in the main text.
